# “Not Designed for Us”: How Science Museums and Science Centers Socially Exclude Low-Income, Minority Ethnic Groups

**DOI:** 10.1002/sce.21133

**Published:** 2014-09-11

**Authors:** Emily Dawson

**Affiliations:** Department of Science & Technology Studies, University College LondonLondon, WC1E 6BT, UK

## Abstract

This paper explores how people from low-income, minority ethnic groups perceive and experience exclusion from informal science education (ISE) institutions, such as museums and science centers. Drawing on qualitative data from four focus groups, 32 interviews, four accompanied visits to ISE institutions, and field notes, this paper presents an analysis of exclusion from science learning opportunities during visits alongside participants’ attitudes, expectations, and conclusions about participation in ISE. Participants came from four community groups in central London: a Sierra Leonean group (*n* = 21), a Latin American group (*n* = 18), a Somali group (*n* = 6), and an Asian group (*n* = 13). Using a theoretical framework based on the work of Bourdieu, the analysis suggests ISE practices were grounded in expectations about visitors’ scientific knowledge, language skills, and finances in ways that were problematic for participants and excluded them from science learning opportunities. It is argued that ISE practices reinforced participants preexisting sense that museums and science centers were “not for us.” The paper concludes with a discussion of the findings in relation to previous research on participation in ISE and the potential for developing more inclusive informal science learning opportunities.

## INTRODUCTION

The landscape of science learning is broad and ranges from schools to homes to museums, from designed learning environments to chance talks with friends. Within designed science learning environments questions about who participates, how they do so, and why have traditionally been framed in terms of students within schools and universities. In practice and research, attention has been paid to increasing and diversifying the pool of students studying science and pursuing science careers (the science pipeline). Thus there is research on the difficulties experienced by minority ethnic students (Brown, 2004), female students (Carlone, 2003), and low-income students studying science in schools (Calabrese Barton, 1998) that provides helpful information about how school science could be made more equitable. Comparatively less attention has been paid to equity and widening participation in science learning in everyday life, outside the walls of schools, universities, and the labor market.

In the past 30 years, informal or lifelong science education has grown in terms of research, activity, training, and international spread to become a significant feature of the science learning landscape (Bray, France, & Gilbert, 2011; Falk et al., 2012). From Australia to Saudi Arabia, informal science education (ISE) institutions such as science centers, museums, aquaria, and zoos offer opportunities for their visitors to learn about science, understand it, and question it outside school and university curricula and long after they graduate (Packer & Ballantyne, 2002; Zahrani, 2010). In societies where science plays important personal, social, and political roles, ISE offers people valuable experiences that may, among other things, affect the science “pipeline,” public scientific literacy, and public debates about science (Bamberger & Tal, 2008; Falk & Needham, 2011; Lehr et al., 2007). But how accessible are the opportunities ISE provides? In this paper, I explore whether there are grounds to seriously question the claim made by Frank Oppenheimer, the founder of the Exploratorium science center, that “no one ever flunked a museum” (Semper, 1990, p. 52).

Unlike science education in schools and universities, questions of inclusion and exclusion, access, and widening participation have tended to be underresearched and undertheorized for ISE. Research on ISE has instead typically focused on the benefits experienced by existing participants (Falk, 2009; Rennie & Williams, 2006). This is problematic however since descriptive data from the United Kingdom, United States, and international surveys suggest that, at present, ISE institutions are not inclusive spaces. Visitors to ISE institutions come from more affluent, middle-class backgrounds, from ethnically dominant backgrounds (such as White European backgrounds in the case of the United Kingdom), live in urban areas, and visit as part of a school or family group (Bell, Lewenstein, Shouse, & Feder, 2009; Department for Culture Media and Sport, 2011; Ipsos MORI, 2011; OECD, 2012). Research suggests that informal science learning resources cluster around such groups. In other words, those likely to visit a science museum, are also likely to visit other ISE institutions, experience science “shows” in their schools and have the opportunity to be involved in after-school science clubs (Falk et al., 2012). As a result, research on ISE has concentrated around particular social groups and may not apply more broadly across diverse populations nor offer many clues about how to broaden the appeal of ISE.

While there are no specific data on patterns of nonparticipation, these can be inferred from visitor data, which suggest that people unlikely to visit ISE institutions may come from working class or low-income backgrounds and minority ethnic backgrounds. Those living in rural areas, not involved in school education, and living away from their families may also be less likely to visit (Department for Culture Media and Sport, 2011; Ecsite-UK, 2008). The reasons behind such partial patterns of participation are not fully understood.

To date, social exclusion from ISE has been primarily framed in terms of “barriers,” which prevent certain people or groups from visiting ISE institutions (Bell et al., 2009; Ipsos MORI, 2011). Such barriers include the structural barriers of cost or geographic distance and attitudinal barriers such as lack of interest in science (Falk, Storksdieck, & Dierking, 2007; Ipsos MORI, 2011).

On the one hand, the barriers model appears to make sense, of course people living in poverty struggle to afford a science center visit. On the other hand, the barriers model does little to explain how barriers might overlap (e.g., combinations of poverty and lack of interest) or the resilience of demographic patterns in ISE participants despite attempts to change such patterns. For example, following a barriers approach financial “barriers,” in this case entrance fees, were removed from a number of museums in the United Kingdom, including the Science Museum and the Natural History Museum, through government subsidy in the 1990s. Visitor numbers rose dramatically. Analysis of who these visitors were suggests, however, that the removal of entrance fees did little to diversify museum visitors; existing visitors simply visited more often (Ipsos MORI, 2003). Thus the removal of a financial barrier had little effect on who did and did not visit such institutions and suggests that the barriers perspective actually offers little insight into why and how social exclusion and nonparticipation operate.

The “barriers” approach has been criticized as assimilationist for requiring participants to change to fit institutions, privileging dominant knowledge and practices, and pathologizing others (Bell et al., 2009; Lee, 1999; Levitas, 1998; Yosso, 2005). This is a damaging practice because, as Rahm and Ash (2008, p. 60) suggest “many ethnically and linguistically diverse youth from low-income backgrounds are positioned as ‘problems’ by the [science education] system, which then results in the positioning of self as outsiders.” Instead of changing patterns of participation, taking a barriers perspective instead risks perpetuating a cycle of social exclusion and nonparticipation (Dawson, 2014).

Moving away from an assimilationist perspective may not be straightforward. An assimilationist perspective to some extent protects the powerful social roles played by science and technology in contemporary societies. For example, research on ISE institutions suggests science is represented as a form of authority, or truth, in ways that avoid alternative interpretations, are heteronormative and male dominated (Gieryn, 1998; Levin, 2010; Macdonald, 2002; Machin, 2010; McNeil, 2007). Encountering such representations of science may be problematic for people from particular groups or backgrounds, as research on science education in schools has found (Lee & Buxton, 2010; Roth & Calabrese Barton, 2004).

Research in the United States and Canada suggests that social positions, such as gender, ethnicity, class, or age, may play a more important role in ISE experiences than the “barriers” model suggests (Aikenhead, 2002; Bell et al., 2009; Fenichel & Schweingruber, 2010; Rahm, 2010). For example, research on ISE in the United States suggests there is a gender bias in parent behaviors and in exhibit design that favors boys (Borun, 1999; Crowley, Callanan, Tenenbaum, & Allen, 2001; Dancu, 2010). Similarly, North American studies of families from Latin-American backgrounds found ethnicity played a key role in ISE institution visit experiences. The families found science museums were unwelcoming, expensive, difficult in terms of the language used, and felt the activities provided were unimportant and irrelevant (Ash, 2004; Garibay, 2009; Rahm, 2010). Outside the United States and Canada, however, social exclusion and nonparticipation in ISE has received less research attention.

Studies on school trips to museums and science centers have argued that such trips benefit children from socioeconomically disadvantaged backgrounds (Hooper-Greenhill, Phillips, & Woodham, 2009) and can affect some children over a year after their visit (Bamberger & Tal, 2008). From this perspective, school trips to ISE institutions may provide some leverage for shifting patterns of participation in ISE. In the context of social inclusion and exclusion from ISE, such interpretations warrant careful consideration. For example, school trips have been found to be complicated for ISE practitioners, teachers, schools, and students in a variety of ways and in more than one country (Anderson, Kisiel, & Storksdieck, 2006; DeWitt & Osborne, 2007; Hofstein & Rosenfeld, 1996), raising questions about the longer term effects of such visits on lifelong participation in ISE. School trips to museums and science centers have a long history in some countries whose patterns of participation in ISE remain partial. Given the issues discussed later in this paper, I suggest the influence of school visits on social inclusion in and exclusion from ISEs is considered cautiously. These studies do, however, highlight the need to go beyond the barriers model and to develop a better understanding of how and why social exclusion and nonparticipation in ISE operates empirically and theoretically.

This paper challenges conventional notions of exclusion in ISE, unpacking well-worn theories of exclusion based on the barriers perspective. The study presented in this paper explored in detail how social exclusion from ISE worked in practice from the perspective of people from disadvantaged social positions, drawing on data collected in the United Kingdom. In this paper I focus on experiences of nonparticipation in and exclusion from ISE to ask the following research questions: Why do people from underprivileged backgrounds not visit ISE institutions and what happens when they do?

Inherent in social research on experiences of exclusion is the risk of essentializing those involved and contributing to problematic constructions of “nonparticipants,” “nonvisitors,” “the excluded,” “other,” or “underprivileged groups” (Cribb & Gewirtz, 2005). The problems and politics of identification and representation are no small matter and, as Young has argued, “all systems and institutions of representation group individuals according to some kind of principles, and none are innocent or neutral” (Young, 2000, p. 143). Exploring nonparticipation and representation in terms of who can and cannot access resources and what is at stake in questions of participation is important, but risky. In this paper, I have tried to balance describing, analyzing, and representing people and their specific experiences against the problems inherent in such work, not least contributing to constructions of “others” (Young, 2000). This paper provides concrete examples of social exclusion from ISE and suggests that understanding experiences of difference, discomfort, and inaccessibility are crucial for creating more inclusive ISE practices.

## THEORETICAL BACKGROUND

This paper uses theoretical tools drawn from Bourdieu to explore social exclusion and nonparticipation in ISE. Bourdieu suggested social disadvantages are produced and maintained by the relationships between peoples’ behaviors and dispositions (their habitus), the resources available to them (their capital), and the organization or structure of the situations people find themselves in (the field) (Bourdieu, 1998). I draw on theoretical tools developed by Bourdieu because they enable a study of practice in a particular setting to include people's attitudes, behaviors, available resources, and the structure of the setting in question, paying attention to issues of power and disadvantage.

Bourdieu described habitus as the result of people's experiences, the “conditions of existence which, in imposing different definitions of the impossible, the possible, and the probable, cause one group to experience as natural or reasonable practices or aspirations which another group finds unthinkable or scandalous, and vice versa” (Bourdieu, 1977, p. 78). I use the concept of habitus to try and understand the ways in which people recognize their worlds, such that without thinking they know what to pay attention to, what to overlook, or what to do in particular situations. Bourdieu argued that through habitus, experiences of disenfranchisement are mirrored in “different sets of dispositions with regard the social games that are held to be crucial to society” (Bourdieu & Wacquant, 1992, p. 172). Thus people become disposed against activities they feel are of little use or relevance to them, for example, choosing not to study at university because they perceive it as unnecessary for the kinds of jobs they expect to do in the future. This “system of dispositions” (Bourdieu & Passeron, 1990, p. 67) is, however, dynamic. Habitus affects new experiences as much as it is influenced by earlier experiences and while resilient is not fixed. Thus, while a person's habitus structures their behaviors, assumptions, and lifestyle, it could shift over time as the result of transformative experiences.

Bourdieu argued that patterns of habitus can be identified within social groups whose experiences may be shared. People exposed to similar settings, attitudes, and practices share a similar habitus, because, as Bourdieu and Waquant argue, these experiences are “already predefined by broader racial, gender, and class relations” (Bourdieu & Wacquant, 1992, p. 144). Thus, Bourdieu argues, people in similar social positions share similar experiences, which in turn produce a similar habitus, resulting in what Bourdieu and Passeron described as “class habitus” (Bourdieu & Passeron, 1990, p. 204). For Bourdieu and Darbel (1990), the conceptual art museum was the preserve of the upper classes and would only ever have a limited appeal to working-class people who may perceive such institutions as irrelevant for them. If this is the case with an art gallery, to what extent is this also the case for a science center?

Scholars have developed the concept of habitus further to encompass the practices and assumptions made by institutional staff, which while contested, is a useful tool for exploring experiences in institutions (Atkinson, 2011; Reay, 1998). Here I draw on the concept of institutional habitus to understand how ISE practices treat different kinds of visitors, often in ways that are not explicitly defined, but result from assumptions made by staff or expectations based on how practices are usually carried out within a particular institution.

This paper uses Bourdieu's concept of capital as well as habitus. Bourdieu argued that people manage the situations they are in by using resources such as knowledge, skills, social connections, or money, which he called forms of capital, alongside their “feel for the game” (Bourdieu, 1998, p. 83), or habitus. I draw on the concepts of cultural capital, linguistic capital, symbolic capital, social capital, and economic capital to explore peoples’ experiences of visiting ISE institutions. These forms of capital correspond to the tools or resources people use or generate through certain behaviors in specific fields, in this case in ISE.

Cultural capital can be understood as the kinds of knowledge and skills a person brings to bear on a situation. In ISE, for instance, useful cultural capital could include knowledge of scientific concepts or knowing how to use an ISE institution. Linguistic capital is closely related to cultural capital and frames individual's language competences, whether, for example, they are multilingual, whether they speak with an accent, whether they use certain words or others. Being able to understand and use scientific language such as “hypothesis” or “nucleus” could, for example, be considered as both linguistic and cultural capital. Symbolic capital, in turn, is about the idea or honor or prestige, often encoded into someone's status or taking part in a high-status activity.

Social capital results from the properties someone builds “by virtue of possessing a durable network of more or less institutionalized relationships of mutual acquaintance and recognition” (Bourdieu & Wacquant, 1992, p. 119). In other words, social capital comes from social networks, the colleagues, family, and friends that a person can influence and, in turn, be influenced by. Economic capital, finally, refers to the available financial resources a person can leverage in a given situation. In the context of ISE institutions, economic capital may be involved in paying more money for entry to a special exhibition, buying gifts from the shop, or being able to afford the transport to get there.

In this paper, I also draw upon Bourdieu's concept of symbolic violence. For Bourdieu, symbolic violence lay in the misrecognition of overt exclusion, domination, or inherited advantage. He described it as “gentle, invisible violence, unrecognized as such, chosen as much as undergone” (Bourdieu, 1990, p. 127). For example, a school system where the knowledge that “counts” is that of the dominant groups can be understood to disadvantage students from nondominant groups by rendering their knowledges and practices as “other,” such that those students eventually decide not to pursue higher education, taking themselves out of the system (Bourdieu & Passeron, 1990; Ogbu, 1992; Yosso, 2005; Young, 2000). That such practices obscure the reproduction of inequality is at the heart of their power (Bourdieu & Passeron, 1990). In this paper, I explore where and how habitus, forms of capital, and symbolic violence might be identified in the attitudes and experiences of participants from marginalized social positions and in ISE practices.

## METHODS

This paper draws on a qualitative study that examined how exclusion and nonparticipation in ISE happened in practice by exploring the experiences and perspectives of people from disadvantaged social positions before, during, and after visiting an ISE institution of their choice in the United Kingdom. Identifying individuals and groups as marginalized, underprivileged, or as “nonparticipants” is, as suggested above, not without problems. Participants were recruited from grassroots community groups in a central London borough following a participant profile developed for the study. I acknowledge, however, that group membership, whether organized around ethnic, socioeconomic, or other shared practices or identities, is not necessarily the identifying characteristic it is sometimes presumed to be (Hoggett, 1997; Spencer, 2006). This approach to participant recruitment was based on descriptive data about who might not participate in ISE and was designed to be exploratory rather than representative (Gobo, 2004). As such, the experiences of individual participants are not intended to represent the experiences of their community or people in similar social positions, but to probe perceptions and practices.

Participants were recruited on the basis that their social positions included an overlap between low-income backgrounds, minority ethnic status, and a mixture of ages and genders. This profile was designed to maximize opportunities to explore the different facets of social position and disadvantage in relation to ISE participation. Participants were recruited from a mixture of migratory trajectories. In other words, some participants were recent migrants, new to the United Kingdom, whereas others were more established or born and raised in the United Kingdom. Central London was used as the research site because London has a diverse population, among whom live particularly marginalized communities with an array of ISE institutions on their doorsteps (Sassen, 2001).

Community groups were identified on the basis they were self-organizing, based in Central London, and whose members fit the participant profile developed for this study. A snowball sampling process (where one contact suggests several other contacts) was negotiated with community network “gatekeepers” as starting points. Sixty-eight community groups were approached in this manner, from which four groups ultimately took part in the study. Participants came from a Sierra Leonean group (*n* = 21), a Latin American group (*n* = 18), a Somali group (*n* = 6), and an Asian group (*n* = 13). Notably, more women (*n* = 38) than men (*n* = 20) took part in the visits. Despite analytic attention to gender issues, they did not appear as a distinct theme in this study.

The study took an exploratory, ethnographic approach and worked with participants from four different community groups over a year long data collection period. All fieldwork was conducted by the author (a middle-class, White woman, from a European—French and British—ethnic background, who was seen as a “science insider” by participants). During that year, alongside my participation in community group activities, participants took part in focus groups, interviews, and accompanied visits to an ISE institution of their choice to explore their experiences in situ. This paper draws on these qualitative data which comprise four focus groups, 32 interviews, field notes, and four accompanied visits to ISE institutions (see Table1).

**Table 1:** Numbers of Participants Involved in Each Data Collection Point

Group	Focus Group Participant	Visit Participant	Interviewee
Sierra Leonean	8	14	6
Latin American	12	6	8
Asian	5	10	4
Somali	4	3	4
Total	29	33	22

The year of participant observation formed the basis from which other data collection was carried out. Participant observation happened at community group events to which I was invited, typically at weekends or during evenings, once or twice a month (Brewer, 2000). Following a preliminary phase of agreeing the terms of the study with participants and attending initial community events, a focus group was carried out with each community group. Focus groups were semistructured and discussion guides were used, covering issues such as attitudes toward ISE, expectations of ISE institution visits, and perceptions of ISE institutions, their content, visitors, and appeal.

Interviews were carried out following the focus groups and accompanied visits to explore themes from earlier data collection in more detail. Interviews followed a conversational approach (Hammersley & Atkinson, 1997; Kvale, 1996), but discussion guides were used to follow up on specific issues, such as activities participants had mentioned, questions about particular exhibits, as well as broader questions about experiences or expectations of ISE. While interviews were carried out with each group, these varied more in number due to participants’ availability. Certain participants took on “key informant” roles and were interviewed more than once. At least two participants were interviewed from each group, resulting in 12 postvisit interviews.

Participants chose their visit from a list of ISE institutions available in London during the research period and their own suggestions. Accompanied visits have been used in museum studies to explore visitor experiences in detail (Hooper-Greenhill, Moussouri, Hawthorne, & Riley, 2001). Such visits are a specific form of participant observation where a researcher takes part in an activity with others to observe and document events (Hammersley & Atkinson, 1997). The accompanied visits were not positioned as interventions with measurable outcomes or as comparative studies, but rather as tangible experiences for participants to engage with.

Three groups (the Sierra Leonean group, the Asian group, and the Latin American group) chose to visit science museums with exhibitions of natural history objects, “hands-on” exhibits, and exhibits of living plants and animals. The Somali group decided to visit an interactive science center with computer-based exhibits and audio-visual “shows.” Three different sites were visited, since the Sierra Leonean and Asian groups independently decided to visit the same museum. Visits lasted between 2 and 5 hours. In each case, transport and refreshments were provided (see Table2 for an overview of the visits).

**Table 2:** Overview of the Accompanied Visits

	Visit Participant's Gender			
Community Group	Female	Male	Age Range	Group Type in Visits
Sierra Leonean (*n* = 14)	11	3	Five under 16 Three in their 40s Six between 60s and 75s	Two families + community group members
Somali (*n* = 3)	3	–	Two in their 20s One in her 40s	Community group members
Asian (*n* = 10)	8	2	Two in their 30s One in her 40s Two in their 50s Five between 60 and 75	One family + community group members
Latin American (*n* = 6)	5	1	Two under 16 Two in their 20s Two in their 40s	One family

The focus groups, interviews, and all participant observation except the four visits took place in community venues. The focus groups, visits, and all but one of the interviews were documented using field notes, multiple audio recordings, and photographs as appropriate. Transcripts were anonymized, and pseudonyms chosen privately by participants were used. The analysis was carried out in two steps. The first phase of analysis took place during data collection; data were grouped into categories and regrouped (or coded) as more data were added. Data were coded using the Nvivo software to categories and index data following an iterative, thematic approach, which requires what Kitzinger describes as an “interpretive leap” (2004, p. 138). For the purpose of data analysis, the methods of constant comparative analysis and deviant case analysis were used to categorize data and interrogate themes (Hammersley & Atkinson, 1997; Miles & Huberman, 1994). Data were also analyzed in relation to one another, thus there was analytic triangulation of audio recordings, photographs, field notes, and follow-up interviews. At the end of this stage, the themes from the initial data analysis were discussed with participants which provided opportunities for them to question, oppose, or further interpret the data (Brewer, 2000).

The second stage of analysis involved examining the themes identified in the data working with conceptual tools from Bourdieu, in particular habitus, capital, and symbolic violence. This theoretical framework was selected because, as argued earlier, these concepts are useful for attempting to understand the complex relationships between ISE practices and structures, power and disadvantage, as well as people's behaviors and attitudes.

The concept of habitus was applied to the data to map the attitudes, expectations, and logics that participants used to frame their views of ISE and their own participation in such institutions. Drawing on data from across the whole year, patterns of habitus, as well as potential shifts or changes in habitus were analyzed. The roles of different forms of capital, in particular cultural, linguistic, symbolic, social and economic capital were analyzed. Analytic attention was given to the place of capital, how different forms of capital were valued by participants and by the ISE institutions visited, changes in capital (and which kinds of capital changed), and the kinds of capital needed when visiting an ISE institution. The data were also analyzed using the concept of symbolic violence to explore the roles of capital, habitus, and practice in relation to one another and in relation to questions of power. The findings of this analysis discuss, in what follows, participants’ prior views and expectations of ISE, their ISE institution visits, and their reflections after those visits, with examples used to illustrate particular themes and to show divergence or developments from concepts applied in the analysis.

## ANALYSIS AND FINDINGS

### Assumptions and Imagined Spaces: Expectations of ISE Institutions Before the Visits

Across the four community groups, participants’ prior attitudes toward ISE ranged from disinterest, to anxiety, to confusion and rejection. In terms of their behaviors and practices, few participants had visited an ISE institution before and such visits were infrequent. In every group, participants knew little about ISE institutions—where they were, what they were for, or that in London many were free to enter. ISE institutions were often conflated with tourist attractions in general, such as the London Eye or Madame Tussauds. These ideas contributed to participants’ views of ISE as expensive and of limited value to them, their families, or communities. For example, for Maria, from the Latin American group, there was little apparent difference between a science museum and the London Dungeon, a theme park she knew to be prohibitively expensive for a family of six.

Participants’ shared sense of confusion about ISE, and the associated practice of not-visiting ISE institutions across all four groups suggests a common habitus, or disposition, toward (or in this case, against) ISE. Bourdieu argued that habitus is a product of social position that “retranslates the intrinsic and relational characteristics of a position into a unitary lifestyle” (1998, p. 8). In this sense, visiting ISE institutions was not part of participants’ lifestyles.

Participants described their reasons for not usually visiting ISE institutions in terms of their expectations and assumptions, as well as logistics. Running alongside questions of cost, location, and a lack of information, was a sense of alienation. In other words, in every group some participants described visiting ISE institutions as inconceivable as a result of the perceived social distance between ISE institutions and their community. In the words of Fatimata from the Sierra Leonean group during a focus group:

If you ask me, “Fatimata are you going to come down [to a science center],” I'll tell you “No, there's not going to be anyone like me there, what's wrong with you” (laughter from Fatimata and group), but that's my perception of how I think about things

For Fatimita visiting a science center was laughable and unthinkable due to the social distance between her own community on the one hand and her perception of science center visitors on the other. Filtered through the lens of Fatimata's habitus, ISE institutions were seen as places where “there's not going to be anyone like me there,” such that visiting was understood as “wrong.”

Fatimata's account suggests a mismatch of habitus between Fatimata, her friends, family, and community and ISE institution visitors. To borrow from Bourdieu: “the homogeneity of habitus is what—within the limits of the group of agents possessing the schemes (of production and interpretation) implied in their production—causes practices and works to be immediately intelligible and foreseeable, and hence taken for granted” (1977, p. 80). In this sense, Fatimata's habitus does not pave her way into ISE institutions, but rather works to steer her away from them in ways that are taken for granted by herself and her friends.

The perception of ISE as “not for people like me” was perceived in cultural terms, which were implicitly classed and racialized by some participants. In the interview extract below, Thomas from the Sierra Leonean group describes his views about ISE by talking through his notion of an imagined museum that does not fit with his own “culture,” usual behaviors, or knowledge:

Well in a way it's like, imagine going to a museum, you don't imagine it being very comfortable, and going and what makes you comfortable, like maybe going in a hoody and just … people think that you have to go there a certain way, or do a certain thing when you get there or maybe like, it doesn't fit in with your culture kind of thing, you know what I'm saying? Like, could you go there with a Supermalt and just chill and just look at, you know what I'm saying? Maybe people think you have to go there and behave a certain way or have a certain amount of background knowledge already, so maybe that holds people back.

Thomas's imagined ISE institution was unwelcoming to people like him. His anxieties were projected into this imagined institution, particularly in terms of uncertainty about what might constitute appropriate conduct, that “you have to go there and behave a certain way or have a certain amount of background knowledge.” What Thomas described was his awareness that he does not have what Bourdieu called “the feel for the game” (1998, p. 80). Thomas was concerned about being the wrong kind of person for his imagined ISE institution, particularly in terms of embodied habitus (having the wrong clothing, food, and behaviors) and lacking the prerequisite cultural capital (his concerns about background knowledge) (Bourdieu, 1990).

Here Thomas draws on his sense of the differences between “high” and “low” cultural practices, tastes, and class in terms of the forms of knowledge and behaviors that he believes would be more or less valued within an ISE institution (Bennett et al., 2009; Bourdieu, 1984). As Skeggs has argued, “cultures are valued differently depending on who can deploy them as a resource” (2004, p. 173). Thomas's description of an ISE institution highlights his expectation that his culture, knowledge, and practices will not be useful to him in an ISE institution and may instead work against him. The extracts from Thomas and Fatimata can also be interpreted as not only suggesting they are aware that they do not “fit” well within an ISE institution but as an implicit critique of such institutions. In this sense, the irrelevance of the content, values, and behaviors of ISE institutions to the lives of Thomas and Fatimata is marked as “wrong” rather than their own cultures or practices acting as “barriers.”

Not all participants were as clear as Thomas or Fatimata about their sense of discomfort and disposition against ISE, but in all four community groups, participants talked about ISE as something they did not do, nor a practice they intended to pursue. In the extract below from a focus group, Kirin and Sarasa from the Asian group discuss with me how close Sarasa lives to a particular ISE institution without ever having visited:

Sarasa: I'm living nearby there, but I've never, I haven't seen the place
Kirin: you pass it by
Emily: so how long have you lived near there?
Sarasa: ten years, … still I haven't seen the place
Emily: what about the park, do you ever go in the park?
Sarasa: it's a lovely park, I can see it by from the car, when we pass by
Kirin: oh yeah, so you're very near to it then

This extract suggests geographic location and a lack of knowledge about where ISE institutions are or their popularity are not, for some, direct barriers to participation. Indeed, as Sarasa explains, she has driven past this ISE institution for 10 years, she does not visit this museum because she never has and does not intend to. It is simply something she does not do. For Sarasa, habitus creates the conditions for “an individual agent's practices, without either explicit reason or signifying intent, to be none the less ‘sensible’ and ‘reasonable’” (Bourdieu, 1977, p. 79). This example suggests how resilient habitus can be in guiding patterns of behavior and practice; Sarasa does not describe an active dislike of this ISE institution or feeling unwelcome, nor is she confused about how to visit it, she simply does not. This view was common among participants from the four community groups; visiting ISE institutions was “unthinkable” and outside what they would consider to be normal practice for themselves, their friends, and families. Notable in the analysis of the visits that follows is the extent to which participants experiences of ISE institutions in practice supported their preexisting perception that ISE was not for them.

### Language Practices and Inaccessible Learning Opportunities in ISE Institution Visits

A key difficulty encountered by certain participants from each community group during the visits was language use. All three visited ISE institutions relied exclusively on English as their institutional language, with no evidence of materials in alternative languages apparent during the visits. Participants were, as a result, faced with what Bourdieu described as “the imposition of the dominant language and culture as legitimate and by the rejection of all other languages into indignity” (1998, p. 46). Despite the presence of staff and objects, the science museums and science centers interpretive strategies were designed with text, and therefore English reading ability, as the cornerstone of information provision for way-finding signs, cafes, shops, and toilets, as well as exhibit materials. While participants were recruited on the basis that we could communicate with one another in English, this did not mean all participants were fluent readers of English. English was a second, third, fourth, or fifth language for all participants. As a result, language issues significantly affected participants’ visits, restricting their access to information and learning opportunities as well as their ability to navigate the ISE institutions and, therefore, to feel comfortable or welcome.

On multiple occasions participants translated or attempted to translate exhibit text, staff speech, or museum signs for one another. Within-group translation could not compensate, however, for the language barriers participants encountered. At the most basic level, this emerged when some participants were unable to read exhibit texts, as happened in every group. Language was particularly problematic when participants tried to access computer-based exhibits. During the Latin American group's visit, for example, Ignacio tried and failed to help his daughters use interactive computer exhibits. Audio and text instructions were often highly detailed and delivered simultaneously. The instructions for every interactive exhibit the family encountered were only in English, leaving Ignacio to rely on his bilingual daughters for translation and unable to help them.

The most extreme example of language difficulty and exhibit design took place during the Somali group visit to a science center. The overlap of institutional language practices (linguistic capital) with assumptions about the knowledge visitors brought with them (cultural capital) created a learning opportunity that was impossible for the Somali participants to access despite their efforts. The extract below is from Hamiido attempting to use one of the interactive computer exhibits with me, after I had joined her having seen her confusion at her first attempt, and provides some context to understand how learning opportunities were inaccessible:

(A voice from the machine talks again and says “Now it's over to you, remember, you need to keep the conditions right, the humidity, the pH … .”)

Hamiido: how can I know?
Emily: I think it's going to tell us?
Hamiido: yeah?
Emily: – plant cells are growing—so look
Hamiido: this is coming up
Emily: yeah, ph is going down, so, so the temperatures, ok, oh down with the temperature,
Hamiido: this one?
Emily: yep
Hamiido: ok,
Emily: oh, temperature too low, up a bit, ok, ph low, ph up …
Hamiido: Oh, confusing
Emily: I know
Hamiido: yes, this is for scientists, yeah?

To understand what was happening in this “game,” Hamiido needed to understand several scientific terms and concepts: What plant cells are and how they grow, the effect of pH, temperature, water, humidity, waste, and nutrients. This scientific content knowledge was needed to successfully navigate the exhibit and formed part of the cultural capital the science center had designed the learning experience around. Hamiido needed to be able to follow the instructions from the interactive, in English, that were simultaneously verbal and written, with written instructions on more than one part of the large screen at the same time and no way to repeat the information or slow it down. This interactive computer exhibit appeared to be designed for multiple users, with a large screen and several moving components of image and text, which proved difficult to use alone or in a pair. As the Somali participants discovered to their detriment, they literally did not know how to “play the game.”

Bourdieu and Johnson argued that access to the artistic field of cultural production was restricted, “accessible only to those who possess practical or theoretical mastery of a refined code, of successive codes, and of the code of these codes” (1993, p. 120). I suggest a similar practice of restricted access operated within the ISE institutions visited. For instance, in the example above, the learning opportunity designed by the science center implicitly assumed visitors understood a number of codes, not least English language, scientific language, and content knowledge, and how interactive computer “games” commonly found in science museums and science centers worked. Without the linguistic and cultural capital required by these institutions, participants struggled to navigate the institution or access science learning opportunities. The research participants, therefore, faced a tripled disadvantage in the design of the computer-based learning opportunity, since they did could not decipher the “codes” the extent to which participants could *accrue* cultural capital through their visits was limited. Thus ISE practices can restrict access to the field of “scientific” cultural production because they are designed such that only those whose cultural capital is sufficiently aligned with that of an ISE institution can access the science learning opportunities therein and accrue more cultural capital. Such processes involve symbolic violence in that they appear equitable but are not in practice.

### Language Barriers and Feeling Unwelcome in ISE Institutions

Language barriers not only prevented participants from being able to use, understand, or learn from the learning opportunities designed by the ISE institutions visited but also contributed to participants’ broader sense of feeling uncomfortable and unwelcome. Language in this sense played a symbolic role in signaling to participants that they were out of place, defined in the ISE setting in “relation to language” (Bourdieu & Passeron, 1990, p. 116). For example, some Sierra Leonean participants struggled to read exhibit texts but also felt anxious and lost because they could not follow institutional signage and did not feel confident asking for directions in English.

Mama Kamara and Mama Sesay from the Sierra Leonean group concluded, for instance, at the end of their visit that they would not feel comfortable returning to the museum without someone they knew to help them. As they put it,

Mama Kamara: the only problem we have, because, lot of we, some of the people they learn a bit, but some of we no went to school, don't know anything, so if you only take bus to come and see these people here, maybe going inside, getting lost, because you don't know, you don't know how to read, to this way, exit, this that? You can't know, so unless you come with somebody we know, then you come and see the place,
Mama Sesay: because if you come back, you might not want to ask, if you want to be sure, you need to ask somebody, which my way, I want to go out, or I want to go up, or I want to see something and they will show you “go this way,” and when if you want to go out and you don't know, you can ask someone, I want to go out, I'm tired,

Without being able to read signs or ask for directions, Mama Kamara and Mama Sesay felt not only that the whole institution was inaccessible for them, but that they were themselves out of place, anxious, unsure of what to do, and unable to return without support. The institutional reliance on English left Mama Kamara and Mama Sesay with a sense of not being sufficiently educated, linguistically deficient, and profoundly uncomfortable in the museum. This example suggests that language has the power not only to prevent people being able to access cultural capital but to contribute to an impression of ISE institutions as spaces of privilege through dominant language use and as a resource for certain groups rather than others.

The lack of alternative languages and translations in the ISE institutions was noted by several participants. Participants were, however, tentative about suggesting this was an institutional problem, with those in the Latin American and Somali groups commenting on the potential complexity and expense of providing universal translations from the perspective of an ISE institution. As evident in the quote above from Mama Kamara and Mama Sesay, some participants tended to associate linguistic and educational deficiency with themselves, rather than a problem with the museums or the science center. Such processes can be understood as a way in which marginalized groups internalize their exclusion, resulting in hard to recognize symbolic violence (Bourdieu & Passeron, 1990).

Not all participants internalized language problems as their own fault. As Idyl from of the Somali group noted during an interview, the science center was “open to everyone” but did not cater for the linguistic practices of local minority ethnic communities or tourists, pinpointing a key contradiction in the institutional practices of the center she visited. The purportedly accessible institution was, in practice, linguistically structured in ways that made it almost impossible for visitors with limited English language fluency to use. Thus, as these different examples suggest, participants’ relationship to the languages they encountered in the ISE institutions was complex, seen in terms of language “barriers” on their own parts and that of the institutions visited.

Language practices can be understood as forms of capital that delineate the information, culture, and people who are more valued in a particular field (Bourdieu, 1991). In this sense, the exclusive use of English encountered by participants in all four visits suggests that language practices play a powerful role in social exclusion. Bourdieu (1991) suggested that at the heart of language use are questions about which languages are valued by particular institutions and, which groups are therefore, symbolically, valued or not. The way the three ISE institutions used language can, therefore, be understood as symbolically powerful, a message participants’ picked up throughout their visits. In this sense, as Skeggs has argued, “institutional apparatus exists to institutionalise entitlements” (2004, p. 151), which, through their visits, participants experienced in reverse, being instead marked linguistically not only as “other” but as unentitled.

### Interactions With ISE Staff: More Hindrance Than Help

The staff whom participants met in the ISE institutions did not always play a helpful part in their visits. ISE visitor support staff have many roles, from front-of-house staff to “explainers” at particular exhibits. Staff in visitor support positions are thought to provide valuable interpersonal connections between visitors and an ISE institutions, to help visitors engage with exhibits, scientific content, and to facilitate their enjoyment and learning in a museum or science center (Fenichel & Schweingruber, 2010). While interactions with staff varied across the four visits, problematic experiences with ISE staff were striking. During the Latin American group's visit, for example, staff repeatedly asked participants not to touch the live exhibits. This was repeated several times until Maria became offended, since her daughters were being careful not to touch the exhibits. At the end of their visit, the group was offended again as a security guard asked them to leave at 5.30 pm, evacuating them from the museum café, which they thought closed at 6 pm. Such findings echo research by Rahm and Ash (2008) that suggests interactions with members of staff play a key role in making visitors feel welcome, comfortable, and valued in ISE institutions, or not.

Interactions between the Somali group and the science center member of staff who facilitated their session at the interactive science center were particularly problematic. The session had to be booked in advance, thus the institution knew that the group were female and from a Somali community group. The staff member assigned to the group for the facilitated session with the interactive computer exhibits was a young man, who introduced himself as coming from a South Asian background and growing up in a poor neighborhood in a large city in the north of England. He described how he had “used education and science to sort of escape, ‘cos I used to be on quite a rough council estate.” The staff member, Deepak, used identity politics to position himself at the start of the session, outlining how he was similar to the participants (disadvantaged neighborhood, minority ethnic background) but also different to them (had a science degree, worked in a science center, and, notably for the Muslim Somali women, male).

While introducing the session and the work of the science center, Deepak drew at length on his “scientific identity” (Carlone & Johnson, 2007). He was, in some senses, evangelical about the power of science education to change lives, telling participants that he worked “directly with young people, trying to sort of inspire them with science, so schools and colleges.” He was, however, less clear about his work outside the school system, saying,

I also do work with the general public, in terms of engaging everyone else to understand science, to be interested in it, the center has several links to the local community, sort of.

Deepak's professional practice appeared, as suggested by how he described his work, to be oriented toward working with young people, particularly school students, who could be duly “inspired” by the science education of the science center, with less emphasis on how such “inspiration” might operate for “everyone else.”

As the visit continued, Deepak struggled to create a comfortable environment for the Somali participants. He asked few questions about the group and instead posed questions about the content of the science center and scientific information that participants could not answer. Deepak's explanations of the interactive computer exhibits were fast and left little space for participants to ask questions or understand what his instructions meant, using what Bourdieu (1990, p. 108) described as the “status authority” conferred upon him as an ISE facilitator. The extract below draws on his explanation of one of the interactives.

Deepak: have you played top trumps before? The card game? Have you? No?
Idyl: no
Deepak: alright so this is a two player game, so you see you've got these categories, you need to pick a number that's going to be higher than hers
Idyl: ok
Deepak: so if the number 10 would be higher, you'd pick the card, but if you think, you're number four is going to be higher than hers, like, for example, carbon dioxide is one of the main air pollutants, so just press on that, so it actually turns out that there's a lot more nitrogen in the air than carbon dioxide, so she wins the card, and it's your turn again (he says to Nadifa)
Nadifa: oooh
Deepak: did you get all that, so go higher than hers, so try not to lean on it remember,
Idyl: ok
Deepak: so you need to lift up your other hand, because you're leaning on it, so pick a number that's going to be higher than hers, you've trumped 700,000, her number's a lot bigger, she has 20 million, so you win the card, cos it's winner stays on,

The talk in this extract is dominated by the staff member. The explanation of what the computer exhibit involved was raced through, paused only to tell a participant off for leaning on the screen. Deepak's explanation relied on the Somali participants understanding scientific content that he did not explain, such as the differences between atmospheric gases and their familiarity with a British children's game. He created few spaces for the Somali participants with their limited English fluency to interact with him beyond the question “did you get all that?” Following this explanation, Deepak disappeared into another part of the room, leaving participants to “play the game” by themselves.

The interactions between Deepak and the Somali participants highlight the assumptions made about visitors that appear to overlook differences between the participants and the “ideal” or “expected” visitor that Deepak was trained for and accustomed to working with. The institutional habitus of the science center and Deepak's facilitation style seemed disposed toward working with young, English speaking, school science students. Given that research suggests English-speaking school students are a core audience for ISE institutions in the United Kingdom, that Deepak's facilitation was geared toward such groups is perhaps not surprising (Falk et al., 2012). Deepak's facilitation style was, however, not helpful for the Somali participants, one of whom later commented in an interview on how confusing she found the interactive exhibits, saying “I didn't know what I was doing, I was just touching, sometimes I was winning really without knowing the reason why.”

Despite his initial attempts to draw on shared low-income, minority ethnic backgrounds, Deepak struggled to support the Somali participants visit. Deepak's facilitation style limited the extent to which the Somali participants could be inspired, “understand science,” or “be interested in it” as he suggested in his introduction. Instead, through his assumptions and questions, Deepak positioned the Somali participants as unknowledgeable about science and as the “wrong” kind of visitor. As Bourdieu and Wacquant put it, “when habitus encounters a social world of which it is the product, it is like a ‘fish in water’: it does not feel the weight of the water, and it takes the world about itself for granted” (1992, p. 127). While Deepak did not appear to “feel the weight of the water” during the visit, the Somali participants did. This example suggests that institutional expectations, practices, and unspoken assumptions can create difficult visit conditions for participants who differ from the embedded expectations about the “imagined” or “ideal” visitors of the science center.

### The Cost of a “Free” Visit

Free admission was standard across all three of the ISE institutions visited for this study, and all participants’ expenses on research days were covered. Nonetheless, many participants found visits expensive in both literal and more hidden ways. There was a widespread assumption across all four community groups that ISE institutions charged prohibitively expensive entrance fees. For example, Abdou from the Sierra Leonean group described feelings of anxiety that focused specifically on that issue during an interview:

Do you have to pay to go to these museums though? Cos that's one thing I always think, that you have to pay, you have to book, ok how can I go there I haven't got a credit card, because maybe they'll not accept the debit card I've got, you know

For Abdou, this was a litany of off-putting issues. His concerns were underscored by twin anxieties about personal finances and the process of paying. Abdou expected ISE institutions to be expensive, and this perception was shared across all four groups of participants.

Notable in all four visits was the extent to which expectations of high costs were met by what participants encountered in the museums and the science center. Participants from each community group noted the implicit costs associated with the visit, such as the expense of travelling to ISE institutions, the price of items in the institutions shops, and the high cost of food and drink. Two of the Latin American participants, for example, expressed distaste at the sight of donation boxes filled with money. Thus ISE institutions were visibly expensive, but visibly rich institutions. The expense of visiting ISE institutions was commented on by Sofia and Flor, sisters from the Latin American group, in an interview after their visit in terms of the effect of cost on visitor demographics:

Sofia: I think people with a higher income as well would be more likely to go,
Flor: what do you mean?
Sofia: ‘cos having a trip out for a day costs a lot more money than you kind of think, even if it's say like initially free to get in, you're talking travel, food costs, going into the gift shop, all of that, so I think as well people who've got higher incomes it's not really an issue, whereas for other people, “oh well, there's however many of us, that's going to add up”

Sofia understood the hidden costs of visiting a science museum as a series of signs telling her the institution was not for “people like her.” For some participants, the difference between their economic capital and the expenses involved in an ISE institution visit was significant, they “added up.” Participants in each group therefore felt the “weight of the water” (Bourdieu & Wacquant, 1992, p. 127) because high costs were all too apparent, and their visit experiences confirmed their expectations that ISE institution visits were prohibitively expensive.

In addition to economic costs, visits were also associated with an opportunity cost by some participants. For participants in every group, the concept of “free time” was contested. Participants described not having free time or leisure time with which to visit ISE institutions or take part in days out. As Bennett et al. (2009) have argued, in contemporary Britain the concept of leisure and choice of leisure pursuits are still marked by class. In each community, group participants described exploitative working conditions and shift-work patterns as nurses, cleaners, shop workers, or security guards, alongside family commitments, with little time for anything else. For example, Kirin, from the Asian group, argued in an interview that people she knew were restricted in terms of being able to use resources such as ISE institutions, saying that “some people, their reach is not so much, they're busy bringing up children, and life is rush, rush.”

Similarly, in another interview, Maria from the Latin American group argued that ISE institution visits were practically impossible because she worked back-to-back cleaning shifts, saying, “but honestly, I'm not joking, you realise the impact of working round the clock, it's so dreadful.” The impact of shift-work patterns and family commitments was felt by many participants to restrict their free time such that it was described as a luxury. This meant that the museum or science center visits were seen as highly unusual and difficult to repeat because of the constraints experienced by participants in their day-to-day lives, echoing previous research that found museums and similar institutions were particularly classed environments (Bourdieu, 1991; Fleming, 2002).

The symbolic violence of such a system, in which possession of time and disposable income are normalized preconditions for successful participation in ISE institution visits, was not without injury for some participants. Maria, for example, raised concerns about visits to science museums in an interview following her visit:

I feel guilty that I'm not doing it all the time … nothing can beat an outing, but you do want to have the cash in your pocket, it's bad enough the little ones saying “I need this,” and as well as that everyone else on the street listening.

Maria framed her guilt in terms of being unable to provide “outings” for her family and a sense of shame at not having “enough cash” in her pocket. Although implicit within such a statement is a critique of the costs associated with ISE, the situation is complicated since Maria explicitly blamed herself, rather than the structures and practices of ISE, for not being able to take her family to visit and use ISE resources in museums or other institutions. Such complex and negative feelings lie at the heart of symbolic violence. Thus, as Bourdieu suggests “symbolic violence accomplishes itself through an act of cognition and of misrecognition” (1992, pp. 171–172), resulting in personalized feelings of guilt rather than placing the “blame” for inequalities on an institution or wider social relations.

### Ain't Misbehaving: Cross-Cultural Meaning Making, Resistance, and Transgression

I have so far discussed the difficulties participants from across the four community groups encountered during their visits, with an emphasis on inaccessibility that brings questions of equity to bear on what is already known about the potential for learning science through ISE institution visits (see, e.g., Allen, 2002; Bamberger & Tal, 2007; Packer & Ballantyne, 2002) and suggests not all visitors are equally able to learn science in ISE institutions. It is, however, important to note that on occasion some participants were able to draw on their own backgrounds to transform an exhibit into something about which they could make sense. Such moments of meaning making are significant given the broader context of inaccessibility experienced by participants because they extend the Bourdieusian theoretical framework used for this analysis to suggest that more inclusive science learning opportunities could potentially be developed.

When they could, participants in each group used their own knowledge, languages, or cultures to connect with the ISE institution content. For example, participants from the Asian group recognized fish from Bangladesh, told stories about fishing as children in other countries, and shared fish recipes. Similarly, in the Latin American group, Ignacio told stories about scorpions in Colombia while looking at scorpion specimens and shared language skills with his daughters while looking at plant exhibits:

Ignacio: yes, yes, I saw
Sofia: that's the cocoa
Ignacio: I saw sometimes in the forest, the fruit, the fruit is for eating, [ochuas], this is the name
Sofia: [ochuas]
Ignacio: that's it

This extract shows how Ignacio, unable to use the English language resources of the museum, still tried to facilitate learning opportunities for his daughters when he was able to do so using his own resources.

These moments can be understood as instances of cross-cultural learning where different contexts and transnational experiences are pulled together and shared, prompted by an exhibit (Aikenhead, 2002, 2006; Roth, 2008). Through these experiences, some participants were able to connect their cultural backgrounds and identities with their ISE encounters. Such moments bridged the gulf between the museum or science center and participants’ habitus. These moments, while rare, enabled certain participants to suddenly recognize something of themselves in the ISE institution, becoming in that instant closer to the “ideal visitor” who could relate to the exhibits, texts, and concepts presented by the museum, which were inaccessible for the majority of their visits.

The most striking example of cross-cultural meaning making occurred during the visit with the Sierra Leonean group, members of which twice danced and sang in response to the objects they encountered. Through their dancing and singing, some of the Sierra Leonean participants briefly disrupted their disempowered relationship with the museum to create connections between themselves, their cultural heritage, and particular exhibits. For example, one participant recognized a bird among a display of several animals, which triggered members of the group to dance and sing next to the exhibit for several minutes. In the extract below, Hawa and Mama Kamara explain the relevance of the bird on display for their community:

Hawa: It's a song for that (points to the bird)
Mama Kamara: For these two birds. When you sing, go to society, people eat for you (sings a bit of the song as illustration), you know that cassava?
Emily: Yeah
Mama Kamara: They boil it, and dry it, the cassava, when they dry it, that's what we call [acolopala]

Seeing the birds prompted some of the Sierra Leonean participants to perform the ceremonial dance for a rite of passage involved with hunting and eating the birds on display. The participants appeared to enjoy themselves, dancing, singing, laughing, and talking. The actions of the Sierra Leonean group can be understood as an interpretive strategy leading to cross-cultural meaning making. Such actions can also be understood as a form of transgression and resistance to the museum, with its objects imprisoned in glass, inaccessible texts, unknown codes of conduct, and incomprehensible language. By dancing and singing, some of the Sierra Leonean participants briefly disrupted the quiet gallery with movement and noise, transforming their experience of inaccessibility and discomfort by reacting to an exhibit on their own terms.

In the weeks that followed the visit, however, some Sierra Leonean participants expressed feelings of anxiety and regret about their behavior in an informal interview about their reflections on their visit to the museum. Without prompting specifically about the dancing or singing, some of the participants described feeling concerned that, as Hawa later put it in an e-mail, they had been “carried away” on the two occasions they engaged with the museum in this way. Such moments of ISE engagement are complex and not unproblematic; although the Sierra Leonean participants had tried to find a role for themselves in the museum, working to be more like the “ideal” visitor, they later felt they behaved in ways that marked them as “other.” Thus, as Bourdieu reminds us, fitting in and reducing social distance is “easier for the dominant than for the dominated” (1977, p. 82). For the Sierra Leonean participants, dancing and singing represented a struggle to change, however briefly, their social position as unknowledgeable and out of place in the museum.

In this sense, to borrow from Trienekens (2002), certain participants were sometimes able to use the ISE institution to build on their community-based cultural capital, which may represent a powerful way for people from socially disadvantaged groups to create relevant cultural and educational opportunities within ISE. However, as others have pointed out (Bennett et al., 2009; Bourdieu, 1984; Skeggs, 2004; Yosso, 2005), forms of knowledge and culture are valued in different ways by societies. Bourdieu argued forms of capital, especially cultural and social capital, sit within a field of power rooted in the dominant practices of a society (1993). While it is important to recognize that research participants were not deficient or without culture, language, or knowledge, it is also important to pay attention to where power lies in the practices of participants and the practices of the ISE institutions visited.

Bourdieu described educational institutions as sites that defend “cultural orthodoxy or the sphere of legitimate culture against competing, schismatic or heretical messages” (1993, p. 112). Power can be traced, therefore, in the knowledge practices of such institutions, and, as hooks (1994) suggests, it is important to trace whose knowledge is privileged and whose ways of knowing matter more. The epistemic practices of the ISE institutions involved in this study privileged certain forms of “scientific” knowledge above other forms of knowledge, as evident in learning opportunities where scientific language, skills, and concepts were a prerequisite for engagement. In contrast, as the experiences of the Sierra Leonean group suggest, little support or recognition was given to facilitate alternative explorations of exhibits, objects, or other learning opportunities within the ISE institutions. Thus, while moments of cross-cultural learning arose during the visits, briefly allowing certain participants to connect with the ISE institutions on their own terms, the extent to which such moments were transformative was limited, leaving some participants feeling they had “misbehaved.”

### A Nice Day Out: The Social and Symbolic Capital of ISE Institution Visits

Despite moments of cross-cultural learning, participants in each group rarely reflected on their visits in terms of learning, but associated them with social benefits instead. While learning is evidently an important part of a visit to any informal *education* institution, ISE institutions have been found to offer valued social opportunities for families and friends to spend time together (Ellenbogen, 2002) as well as for meeting new people (Lehn, Heath, & Hindmarsh, 2001).

Participants’ reflections on their visits echo this previous research and suggest that the visits were, for many, primarily associated with social benefits: spending time with family or friends and sharing food and new experiences. Spending time together also contributed to participants’ social capital within their groups (Bourdieu & Wacquant, 1992). As Maria from the Latin American group put it, “it was lovely to go out as a family and have a focus … they all felt it was a special treat.” For others, such as Idyl from the Somali group, being with friends alleviated the “boring” elements of the visit, she concluded that “we were more enjoying the company and that makes it more exciting than what we saw in there.” More cynically, such extracts can also be understood in the context of visits where participants struggled to understand or access the other aspects of the visits. As such, a focus on the more accessible elements of the visits**—**the company of other participants, often friends and family from the same community group**—**may represent an obvious focus for reflection.

While many participants enjoyed spending time with people they already knew, there were few cases of any participants interacting with people outside their group during the visits to create social links to people beyond their own community group. While ISE institution have been found to offer opportunities to meet new people, build new relationships (Meisner et al., 2007), and even operate as a safe third space for diverse societies (Golding, 2009; Gurian, 2001), the findings of this study suggest this was limited for participants.

In each group, most participants relied on within-group bonding as social capital rather than benefitting from bridging social capital during the visits by building relationships with new people, whether ISE staff or other visitors (Putnam & Goss, 2002). During the Asian group visit, an hour-long, object-handling workshop provided a rare opportunity during the visit for participants to spend time with someone new and therefore to develop bridging social capital. Despite their positive views of the workshop, the reflections of participants from the Asian group, such as Kirin's below, suggest that bridging social capital was out of reach for them:

Kirin: the workshop, workshop, was wonderful I think, the thing that most fascinated me was the elephant tooth, so big and heavy, I can't imagine that you know. I still remember her saying, that animal thing, is it a cat that people wear for good luck? On their shoulders, with the face …
Emily: so what was it about that workshop that made it stand out, that made it seem special to you?
Kirin: yes, the people who were trying to show us everything I think were wonderful, very intelligent people, they have interest in these things, and it was it was different from everyday life, what things we see.

Notable in the extract above is how Kirin differentiated between herself and the ISE staff. She emphasized the differences between herself, people she knew, and their daily lives versus the unusual, special objects, and intelligent staff at the museum. This particular staff-led experience was positive in several ways for Kirin, but a sense of social distance outweighed any sense of building bridging social capital with new people or disrupting the power relationships between herself and the museum. In line with the analysis of participants’ habitus discussed in this paper, for Kirin there was a significant distance between herself and museums as “wonderful,” staffed by “very intelligent people.” Kirin's perception of difference and distinction echoes the social distance between the Somali participants and their facilitator with his “science identity,” in ways that are similar to findings elsewhere about the tensions involved in how people negotiate their relationships with science (Archer et al., 2010; Carlone & Johnson, 2007).

A sense of distinction and high status was, however, at the root of why some participants valued the ISE institution visits and were able to generate social capital outside these institutions afterward. As well as building bonding social capital within the family or community group through a shared experience, some participants later used their visit as a social resource. For example, after the Sierra Leonean visit, Hawa described how one of the participants, who felt unwell throughout the visit, “rang her children straight away to tell them that she'd been to a museum.” Similarly, Abdou from the Sierra Leonean group suggested that after the museum visit participants from the Sierra Leonean group would share their visit experiences with their friends and families to “educate other people about what it's like.”

Abdou and Hawa highlight what Bourdieu described as the symbolic value or symbolic capital of the visits (1998). Some participants perceived ISE institutions as high-status institutions, filled with, as Kirin's description of her visit suggests, special objects, and clever staff. Thus ISE institutions were, for some participants, more special because they recognized them as elite resources for other people. For example, in the Latin American group, two of Maria's children, Paola and Valentina, took leaflets they had found at the museum into school the following week. The value of their actions lay in being able to show a teacher (not friends) that such an activity had been undertaken, as Maria explained: “on a Monday presumably the teachers are saying ‘well what news have you got' and they're quite hard pushed to say, ‘well we relax and unwind and watch telly all weekend’ or something.” Maria's comments suggest her daughters were able to use symbolic capital accrued through a high-status museum visit in a mainstream educational setting to show their weekend activities were in line with what their teachers recognised as valuable (Bourdieu & Passeron, 1990; Yosso, 2005). Thus Paola and Valentina were able to increase their social and cultural capital within the school setting and align with a “good student” identity (Archer, 2008).

The symbolic capital associated with ISE institution visits therefore arose partly because of their high status and the unusual nature of the visits in participants' lives. In Bourdieu's words, symbolic capital “appears in the social relations between properties possessed by an agent and other agents endowed with adequate categories of perception” (1998, p. 104). Through the visits to ISE institutions, participants generated knowledge about such institutions and associated practices that enabled some of them, however briefly, to occupy socially valued positions that were different through their involvement in a high-status activity. It is important to note however that the social and symbolic capital they could accrue through the visits was limited.

### The Reproduction of Nonparticipation and Habitus

As might be expected given the visit experiences discussed here, participants in each group felt no more inclined to adopt a practice of ISE institution visiting after the visits than they did before. Reflecting on their visits, many participants commented that returning to the same museum or science center or a different one seemed difficult and unlikely, contingent upon an unrealistic alignment of factors. As Idyl from the Somali group put it:

Idyl: well if I was going with people that we get on, like, then maybe yes (laughs) but if you didn't have anywhere else to go, then maybe yes, just have a laugh and then pretend that we have visited somewhere today, that could be it
Emily: but if there were other places to go?
Idyl: then I wouldn't put that first (laughs)

Idyl suggested she would only return to the science center if there was nowhere else to go in London with her friends, in other words, that it was extremely unlikely. In contrast to Mama Kamara and Mama Sesay from the Sierra Leonean group, who described being unable to return to a science museum without support, Idyl described an active decision not to return. The extract suggests Idyl's habitus, in particular, her disposition against taking part in ISE and participating in science learning activities beyond schooling was reinforced, rather than transformed or disrupted, through her visit. Ultimately she concluded that ISE institutions were “not designed for us.”

Participants from every group described a range of off-putting factors in terms of visiting an ISE institution again, but being unlike other visitors played an important part in feeling that the science museums and science centers were not for them. Several participants commented on not seeing other visitors like themselves. As Maria, from the Latin American group explained, retelling her daughter Flor's comments after the visit:

she was saying “look at all the people going in mum, they're all white, middle class and wealthy, aren't they, there's not many people I was at college with here.” She made that observation and a lot of it is the money thing, being able to afford it, even though supposedly it's free.

In pinpointing the ethnicity and class status of the “ideal” visitors, Maria and Flor noted a visible difference between themselves, their family, and friends on the one hand and ISE institution visitors on the other. ISE institutions were for visitors who were “white, middle-class and wealthy.”

A second key way that the adult participants from each group distinguished between themselves and ISE institutions was in terms of their age: They felt ISE was for children. For example, Idyl concluded in an interview after her visit that the science center was “maybe kind of childish.” Idyl was not along in drawing such conclusions. Similar views were expressed in all four groups after the visits. In a postvisit interview, Mrs. Mallick, from the Asian group, described the “ideal” ISE institution visitor as children, saying “a museum is more for them.” Similarly, Mr. Bhakta suggested ISE was designed to support science students, “well it's just for the young children who study the science, to get information on a lot of things.” Finding that science museums and science centers appeared to be designed not only for visitors proficient in English language and scientific concepts, with sufficient money and time to visit, but were places for children, was particularly off-putting for several participants.

The practical knowledge the participants derived from their visits (such as what happens in a science center or museum, who else is there) aligned with their prior expectation and habitus, or their “feel for the game” (Bourdieu & Wacquant, 1992, p. 128). Through their visit experiences, participants across all four groups confirmed that ISE institutions were not designed for them. Bourdieu argued that habitus is an “open system of dispositions that is constantly subjected to experiences, and therefore constantly affected by them in a way that either reinforces or modifies its structure” (1992, p. 133). The analysis of the visits presented here suggests the visits served to reinforce participants' dispositions against visiting rather than modifying them.

## DISCUSSION AND CONCLUSION

Despite the extensive literature on the benefits of visits to ISE institutions, not least learning about science, developing science career aspirations, and enjoying science as part of culture with family and friends (Ellenbogen, 2002; Falk et al., 2007; Lehr et al., 2007), the analysis presented here suggests these benefits are not equally available to all and that ISE institutions visits may reinforce social disadvantages for some visitors. In other words, that people can “flunk” museums. This study used a theoretical framework drawn from Bourdieu (1992, 1998) to demonstrate how difference is performed in ISE institutions and found that social exclusion and nonparticipation are part of a complex system and work hand in hand to reproduce disadvantages. As such, the research has outlined the social, economic, and cultural power relations and assumptions ingrained in ISE practice and suggests a “barriers” approach is insufficient for understanding the complicated processes involved in social exclusion from ISE.

In this paper, I explored the expectations and practices of participants from low-income, minority ethnic backgrounds from four different community groups in relation to visiting two science museums and a science center. The study disrupted participants' usual practice and instigated group visits to ISE institutions. But, as discussed, the visits were structured by social inequalities such that participants in every group experienced ISE institutions as unwelcoming places where their knowledges, practices, and selves were “othered” (Young, 2000). While it may be unreasonable to expect one visit to a museum or science center to change long-held, preexisting dispositions against participation in ISE, notable in this analysis was the extent to which visits seemed to confirm choices about nonparticipation. Ultimately, the visits reinforced participants preexisting dispositions against ISE and visiting ISE institutions.

This study was designed as a qualitative, exploratory study following four community groups using an ethnographic approach. As such, it is important to note the limitations of contextual data of this kind. For example, the experiences of the Somali group involved in this project may not apply to other Somali community groups, other minority ethnic groups, other low-income groups, or even this same group at a different point in time. Similarly, this study was limited by a focus on experiences of science in the context of nonparticipation in ISE and four specific visits to ISE institutions. Thus the broader context of how participants experienced science and science education in the rest of their lives is beyond the scope of this research, although it would represent an important area for additional research.

Instead, the explicit aim of the study was to explore social exclusion from ISE in practice and in detail, foregrounding the perspectives of those who did not usually use ISE institutions to provide examples with which to develop theories and discussion around exclusion and ISE. As such, this paper provides theoretically framed, empirical evidence about how social exclusion from science museums and science centers happens in practice, from which lessons can be learnt for those working in ISE practice, policy, and research.

While the findings of this paper cannot offer a conclusive answer to the question of how social exclusion affects attitudes toward and experiences of ISE for everyone, the paper points toward some significant issues. A key conclusion of this study is that is that ISE practices were structured in ways that “othered” those who were not “ideal” visitors. This prevented “othered” visitors from accessing the science learning and engagement opportunities designed by the institution. This finding is perhaps not surprising given the research on social exclusion from cultural and leisure pursuits which suggests such activities are strongly marked by class and ethnicity (Bennett et al., 2009). Similarly, research suggests school science experiences are marked by social position and power in ways that disadvantage those from minority ethnic backgrounds, female students, working-class students, and students living in poverty (Archer et al., 2010; Brown, 2004; Calabrese Barton, 1998; Carlone, 2003). The finding of this paper echo such conclusions; attitudes toward and experiences of ISE were marked by class and ethnicity.

Across the four visits, ISE practice was based on problematic assumptions about the capital that participants could bring to their visits, notably speaking and reading English, understanding scientific terms and concepts, available financial resources, “free” time, and familiarity with ISE institutions. This finding resonates with research from Canada and the United States where language mismatches between ISE institutions and minority ethnic visitors were found to be significant in terms of their access to information and their ability to learn or feel comfortable in ISE institutions (Ash, 2004; Ash & Lombana, 2011; Garibay, 2009; Rahm & Ash, 2008; Yalowitz, Garibay, Renner, & Plaza, 2013). While research by Crowley et al. (2001) and Dancu (2010) found gender differences structured the visit experiences of families in ISE institutions, this paper suggested that socioeconomic status and ethnicity are as important and unpacked how and why this was the case.

Symbolic violence was the cornerstone of educational and cultural legitimacy in the ISE institutions visited, working subtly to foreground dominant cultural practices, knowledges, and values while not recognizing or rendering illegitimate the cultural practices, knowledge, and values of nondominant groups. This finding suggests that ISE reproduces rather than disrupts social disadvantages in ways that were similar to other education systems (Bourdieu & Passeron, 1990). Thus in visiting ISE institutions the research participants risked their own cultural backgrounds and practices being made irrelevant in the face of the knowledge practices of ISE. The symbolic violence was made all the more powerful by the symbolic and social value that some participants themselves placed on museums and science centers as high-status institutions, thereby recognizing the authority of ISE to determine which behaviors and knowledges mattered, and, ultimately, which visitors mattered.

As researchers who have investigated the relationships between power and learning in multicultural settings have argued, it is important to note the relationships between perceived deficits and power (Aikenhead, 2002; Ogbu, 1992; Yosso, 2005). It was not that participants were without capital, but rather that the capital they had was not valued in the ISE institutions. For instance, while all participants were multilingual, being able to speak Arabic, French, Temne, or Spanish was not useful within the ISE institutions. In the face of an institutional habitus that positioned the research participants as far from “ideal” visitors and rendered their capital invisible or useless, it is not surprising that many of the participants' dispositions against visiting were reinforced.

## IMPLICATIONS

This paper represents a move away from a simple, “barriers” model of social exclusion in ISE and offers instead a more theoretically driven understanding of the practice of social exclusion from, and nonparticipation in, ISE. As such exploring the implications, both conceptual and practical, of the argument I have made here is important.

As argued earlier, to date policy and practice around social exclusion from ISE has followed a barriers approach (Bell et al., 2009). My findings show that a simplistic “barriers” model does not sufficiently explain the practices of participants or ISE and can at best provide only a partial understanding of what is at stake in ISE participation and exclusion. While barriers certainly exist, I argue they go deeper than entrance fees and affect more than simple changes in behavior. This study raises important questions about the extent to which social exclusion is a resilient phenomenon, embedded in the habitus of both ISE institutions and those who do not visit them, as well as broader structural features of British society such as poverty, class, and ethnicity. Developing inclusive ISE practices is clearly not straightforward.

My analysis suggests two main implications for those working in ISE. First, that there is a need to recognize and understand the exclusive practices and assumptions embedded in ISE. Second, that “inclusive” informal science learning is not simple but *is* a key issue for science education. Those involved in ISE might consider paying more attention to a broader range of issues when addressing social exclusion, not least examining more closely the assumptions made about visitors and how they might better support cross-cultural science learning opportunities. For example, exploring how to train ISE staff to work with a diverse range of visitors and moving away from the “ideal” visitor profile could be fruitful for research and practice in ISE (see, e.g. Ash & Lombana, 2012).

This paper also questions the institutional reliance on English as the dominant language of ISE institutions in the United Kingdom. The apparent lack of translation or alternative language support is all the more striking given that ISE institutions in the United States (such as the Exploratorium in San Francisco, CA) and in elsewhere in Europe (such as the Experimentarium in Copenhagen, Denmark) provide multilingual exhibit texts in up to three different languages. These comparisons suggest that translation is not beyond the capacity of an ISE institution per say. In multicultural, multilingual cities, language support for visitors may be a crucial step toward inclusive ISE practice.

This paper suggests, as Aikenhead (2002), Roth (2008), and Rahm and Ash (2008) have found, that people whose cultural backgrounds differ from the dominant social group are able to engage in cross-cultural learning, but require some common ground or support to do so. Moments of cross-cultural meaning making have great potential for creating more inclusive science learning opportunities and disrupting the concept of the “ideal” visitor that haunted the ISE institutions. It would be valuable to explore how more inclusive informal science learning opportunities could be developed and facilitated in ISE and cross-cultural meaning making could be a useful starting point for future research.

The findings of this study call for a rethinking of ISE practices in favor of working toward more inclusive science learning opportunities that recognize the needs of a diverse range of people. With this paper, I hope to invite further discussion on the issues of social exclusion, ISE and ways to work toward a more equitable science learning system.
